# Personal

**Published:** 1914-09

**Authors:** 


### PERSONAL.

---

Announcement of removal, travel, and other matters of interest are requested. Please report errors in the listing of any physician in the State and other directories, that we may co-operate with the State Society in securing a correct list.

---

Dr. J. Henry Dowd of Buffalo, by special invitation, will read a paper on the Phosphatic Index, before the Central N. Y. Medical Assn., at Syracuse, in September.

---

Dr. C. A. Vanderbeck of Rochester has moved to 49 Vick Park A.

---

Dr. Roy J. Juhry of Buffalo has moved to 468 Koons Avenue.

---

Dr. E. P. Reimann of Buffalo has moved to 543 E. Ferry Street.

---

Dr. John Leonard Eckel of Buffalo was married to Miss Berenice Long of Chicago, on August 3.

---

Dr. Ross G. Loop of Elmira, who has recently returned from Europe, will limit his practice to surgery.

---

Dr. Douglas P. Arnold of Buffalo, who has recently returned from Europe, has opened an office at 758 Elmwood Avenue, and will restrict his practice to diseases of children.

---

Dr. Frederick Zingsheim of Buffalo spent the latter part of July in the Adirondacks.

---

Dr. Frank H. Ransom of Buffalo spent August in Cotuit.

---

Dr. A. T. Kerr of Ithaca spent the summer at Camp Otter, Dorset, Ont.

Dr. A. L. Beahan of Canandaigua has sold an interest in the Canandaigua Hospital for Physicians and Surgeons to Dr. Harry M. Smith.

---

Dr. Dewitt H. Sherman of Buffalo, returned from the Atlantic coast early in August.

---

Dr. Andrew S. Wiley of Buffalo spent August at Block Island.

---

Dr. Louise Deeckmann of Buffalo spent August at Lake Placid.

---

Dr. Mary Innis Denton, a former resident of Buffalo, visited in Buffalo, during August.

---

Dr. Julius H. Potter of Buffalo returned from the Catskills early in August.

---

Dr. O. S. McKee of Buffalo, toured through the Berkshires in August.

---

Dr. Edward D. Clark of Buffalo was slightly injured August 9, on account of his steering gear breaking so that his auto swerved into an electric light pole.

---

We acknowledge with thanks, the courtesy of Dr. P. L. Alden of Hammondsport, surgeon for Glen Hammond Curtiss, for conducting us to the aviation field and explaining the construction of the America, of Langley's model of airships, for which Mr. Curtiss still has strong hopes of success and for enabling us to witness at close range, the flight of hydro-aeroplanes and of a new model of hydroplane, moved by aerial propellor but without wings, and intended merely to skim the surface of the water at high speed and to serve, in military use, for the rescue of disabled hydro-aeroplanes. Dr. Alden recently made the first professional call in the world by aeroplane, reaching a fracture case 10 miles distant in as many minutes.

---

Dr. Flavel B. Tiffany of Kansas City, aged 68, rejoices in the arrival of a son. In congratulating Dr. Tiffany, we beg leave to boast that our great grandfather had the same experience at the age of 81.

---

Dr. John Parmenter, formerly of Buffalo, residing near Geneva, N. Y., since 1908, will probably represent Geneva at the constitution convention.

